# Geographic Patterns of Genetic Variation in a Broadly Distributed Marine Vertebrate: New Insights into Loggerhead Turtle Stock Structure from Expanded Mitochondrial DNA Sequences

**DOI:** 10.1371/journal.pone.0085956

**Published:** 2014-01-23

**Authors:** Brian M. Shamblin, Alan B. Bolten, F. Alberto Abreu-Grobois, Karen A. Bjorndal, Luis Cardona, Carlos Carreras, Marcel Clusa, Catalina Monzón-Argüello, Campbell J. Nairn, Janne T. Nielsen, Ronel Nel, Luciano S. Soares, Kelly R. Stewart, Sibelle T. Vilaça, Oguz Türkozan, Can Yilmaz, Peter H. Dutton

**Affiliations:** 1 National Research Council Research Associateship Program, Washington, D.C., United States of America; 2 Marine Mammal and Turtle Division, Southwest Fisheries Science Center, National Atmospheric and Oceanic Administration Fisheries, La Jolla, California, United States of America; 3 Archie Carr Center for Sea Turtle Research and Department of Biology, University of Florida, Gainesville, Florida, United States of America; 4 Unidad Académica Mazatlán, Instituto de Ciencias del Mar y Limnología, Universidad Nacional Autónoma de México, Mazatlán, Sinaloa, México; 5 Department of Animal Biology and IRBio, Faculty of Biology, University of Barcelona, Barcelona, Spain; 6 Marine Turtle Research Group, College of Life and Environmental Sciences, University of Exeter, Cornwall Campus, Peryn, Cornwall, United Kingdom; 7 Departamento de Biología, Universidad de Las Palmas de Gran Canaria, Campus de Tafira, Las Palmas de Gran Canaria, Spain; 8 Daniel B. Warnell School of Forestry and Natural Resources, University of Georgia, Athens, Georgia, United States of America; 9 Department of Biology, University of Miami, Coral Gables, Florida, United States of America; 10 Zoology Department, Nelson Mandela Metropolitan University, Summerstrand Campus South, University Way, Summerstrand, Port Elizabeth, South Africa; 11 Projeto TAMAR-ICMBio, Salvador, BA, Brazil; 12 The Ocean Foundation, Washington, D.C., United States of America; 13 Laboratório de Biodiversidade e Evolução Molecular, Instituto de Ciências Biológicas, Universidade Federal de Minas Gerais, Belo Horizonte, MG, Brazil; 14 Adnan Menderes University, Faculty of Science and Arts, Department of Biology, Aydin, Turkey; Natural History Museum of Denmark, Denmark

## Abstract

Previous genetic studies have demonstrated that natal homing shapes the stock structure of marine turtle nesting populations. However, widespread sharing of common haplotypes based on short segments of the mitochondrial control region often limits resolution of the demographic connectivity of populations. Recent studies employing longer control region sequences to resolve haplotype sharing have focused on regional assessments of genetic structure and phylogeography. Here we synthesize available control region sequences for loggerhead turtles from the Mediterranean Sea, Atlantic, and western Indian Ocean basins. These data represent six of the nine globally significant regional management units (RMUs) for the species and include novel sequence data from Brazil, Cape Verde, South Africa and Oman. Genetic tests of differentiation among 42 rookeries represented by short sequences (380 bp haplotypes from 3,486 samples) and 40 rookeries represented by long sequences (∼800 bp haplotypes from 3,434 samples) supported the distinction of the six RMUs analyzed as well as recognition of at least 18 demographically independent management units (MUs) with respect to female natal homing. A total of 59 haplotypes were resolved. These haplotypes belonged to two highly divergent global lineages, with haplogroup I represented primarily by CC-A1, CC-A4, and CC-A11 variants and haplogroup II represented by CC-A2 and derived variants. Geographic distribution patterns of haplogroup II haplotypes and the nested position of CC-A11.6 from Oman among the Atlantic haplotypes invoke recent colonization of the Indian Ocean from the Atlantic for both global lineages. The haplotypes we confirmed for western Indian Ocean RMUs allow reinterpretation of previous mixed stock analysis and further suggest that contemporary migratory connectivity between the Indian and Atlantic Oceans occurs on a broader scale than previously hypothesized. This study represents a valuable model for conducting comprehensive international cooperative data management and research in marine ecology.

## Introduction

Marine vertebrates with cosmopolitan distributions often exhibit high dispersal and weak population structure across large spatial scales. Notable exceptions include groups that exhibit reproductive philopatry despite extensive migrations and dispersal potential, such as salmonids [1], cetaceans [2], sharks [3], and marine turtles [4]. The loggerhead turtle (*Caretta caretta*) is a globally distributed species with a complex life cycle. After leaving their natal beaches, hatchlings swim into major ocean surface currents and may be transported across entire ocean gyres as epipelagic, oceanic juveniles [5]–[7]. Large juveniles often switch to benthic foraging behavior upon recruitment to neritic habitats in the region of their natal beaches [8], although stable isotope and satellite telemetry data suggest that a portion of adults in some populations maintain oceanic foraging behavior [9]–[12].

Females exhibit natal philopatry, returning to nest in the region where they hatched [13]. Properly characterizing female natal homing behavior is important for defining the scale at which rookeries should be managed as demographically self-contained populations with respect to nesting female recruitment. In the absence of direct data on natal philopatry from hatchling marking studies, analysis of maternally inherited mitochondrial DNA (mtDNA) offers a means of indirectly inferring female natal homing and dispersal among nesting sites. Management Units (MUs) in this context are defined as rookeries with significant differences in mtDNA haplotype frequencies [14], and these populations are considered to be demographically isolated with respect to female natal recruitment. The use of mtDNA markers to define MUs for marine turtles is justified given that female natal homing defines reproductive population boundaries [15]. Nuclear gene flow can occur among distinct nesting populations as defined by mtDNA when turtles are admixed on foraging grounds or along migration corridors [15]. This migration-mediated gene flow should not detract from classification of rookeries as independent nesting populations because female recruitment is what sustains rookeries demographically, irrespective of the level of migration-mediated gene flow [15]. Despite potential resolution issues with the use of mtDNA to infer demographic isolation of nesting populations [16], [17], significant differences in mtDNA haplotype frequencies provide a reasonable first approximation for defining MUs until more direct demographic measures become available. Beyond delimiting MUs, rookery haplotype frequencies are also important for providing baseline data to inform Mixed Stock Analyses (MSA), which are used to estimate rookery origins of foraging turtles [6]. Therefore robust genetic data from natal rookeries are critical for assessing connectivity throughout the complex life cycle of this species.

At the global level, nine regionally significant nesting aggregations have been recognized as Regional Management Units (RMUs) based on genetic, demographic, geographic, and oceanographic considerations: 1) Northwest Atlantic Ocean, 2) Southwest Atlantic Ocean, 3) Northeast Atlantic Ocean, 4) Mediterranean Sea, 5) Southwest Indian Ocean, 6) Northwest Indian Ocean, 7) Southeast Indian Ocean, 8) North Pacific Ocean, and 9) South Pacific Ocean with a tenth putative RMU proposed for the Northeast Indian Ocean for which genetic and biological data are lacking [18]. Under the Endangered Species Act, nine Distinct Population Segments (DPSs) were recently designated, and these also recognize broad geographic partitioning that is generally consistent with Wallace et al.’s RMUs [19]. Studies based on a 380 base pair (bp) fragment of the mitochondrial control region have demonstrated genetic partitioning within the Northwest Atlantic [20], Southwest Atlantic [21], and Mediterranean RMUs [22], with at least seven, two, and four MUs proposed in the respective regions. Despite clear indication of genetic structure through significant frequency differences, widespread haplotype sharing across ocean basins has limited the utility of the 380-bp sequences as a population marker in MSA. In particular, haplotype CC-A2 was detected in all rookeries sampled in the Atlantic and Mediterranean except some of the southeastern United States north of Florida and the major Brazilian nesting aggregations [15], [20], [21]. Similarly, haplotype CC-A1 was the most common and widespread haplotype among North Atlantic rookeries [15], [20], [23].

Regional reassessments of population structure using longer control region fragments (760 to 817 bp) and representing previously unsampled rookeries have demonstrated that additional MU designations were warranted [24]–[26]. Newly characterized variable positions have strengthened inferences of independence among rookeries that were already considered demographically isolated through subdivision of shared haplotypes [27], [28], which should in turn improve resolution capacity of MSA. The Southwest Atlantic, Southwest Indian, and Northwest Indian Ocean RMUs have not yet been characterized with respect to the expanded control region sequences, and phylogeographic analyses have been limited to finer scale regional assessments with the expanded sequences. A series of workshops held in 2009–2010 led to the establishment of a working group to bring together data holders and analysts to compile a comprehensive assessment of stock structure and phylogeography for loggerhead turtles in the Atlantic and Mediterranean basins to form the baseline for future MSAs. We accomplished this through synthetic genetic analyses of loggerhead turtle rookeries in the Atlantic Ocean, Mediterranean Sea, and western Indian Ocean combining published expanded control region sequences from regional analyses and novel sequence data from Cape Verde, Brazil, Oman, and KwaZulu-Natal, South Africa.

## Methods

### Ethics Statement

This research was approved by Institutional Animal Care and Use Committees at the University of Florida (201101985) and the University of Georgia (A201201-025-R1). Georgia samples were collected under Georgia Department of Natural Resources permit 29-WJH-13-37. Florida samples were collected under Florida Fish and Wildlife Conservation Commission permits MTP-016, MTP-130, and MTP-135. This work was conducted under SISBIO permit 14122-1 from the Brazilian Ministry of the Environment, and samples were exported under CITES permit 11BR006778/DF. Samples were collected in South Africa under authority of the Department of Marine Affairs permit RES2010-44 and RES2010-55 and exported under CITES permit 106682. Samples were imported into Spain (University of Barcelona) under CITES permits ESBB00601/03-I, TR18080303092, 106126/3423 and 1186. Samples were imported into the United States under CITES permits 13US724540/9 (Archie Carr Center for Sea Turtle Research) and 09US844694/9, 10US844694/9 (NOAA - Southwest Fisheries Science Center).

### Sampling Design and Locations

**Figure 1:**
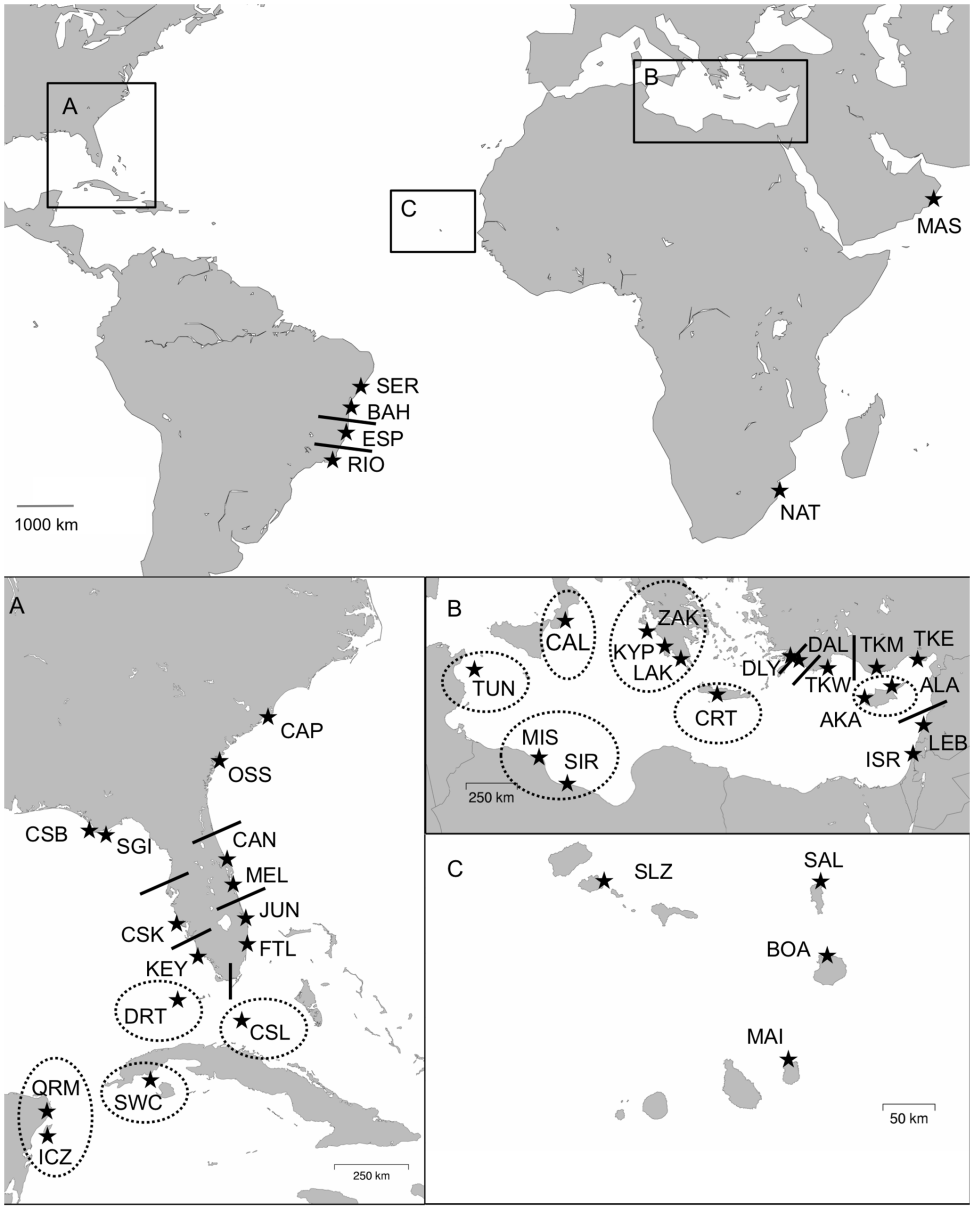
Sample sites for rookery haplotype data for the six regional management units in this analysis. Rookery abbreviations are detailed in Table S1. Solid lines and dashed circles indicate proposed management unit divisions for recognition of demographically isolated nesting populations (including those that were not genetically well differentiated), but do not define precise boundaries.

Haplotype counts representing 380 bp and ∼800 bp control region sequences were taken from the literature or generated from novel samples (Table S1, Figure 1). New samples were collected from Cape Verde, Brazil, and South Africa, and expanded sequences were generated from previously analyzed samples from Brazil, South Africa, and Oman (Table S1). Sequences from a total of 3,486 samples (380 bp haplotypes) and 3,434 samples (760 to 817 bp haplotypes) were analyzed. Primary data was generated in several laboratories from samples collected by different groups and then compiled and analyzed by a consortium of researchers that established the Atlantic and Mediterranean Loggerhead Genetics Working Group through a data sharing agreement. Sampling design and protocols varied among individual studies, but briefly, samples were obtained directly from nesting females via blood or skin biopsy or from loggerhead turtle nests via undeveloped eggshells, blood from emerged hatchlings, or tissue from dead embryos or hatchlings during post emergent nest evaluations (Table S1).

### Laboratory Analysis

Genomic DNA was extracted using standard phenol-chloroform isolation or the DNeasy blood and tissue kit (QIAGEN) following standard protocols. Polymerase chain reaction (PCR) amplifications of an ∼800 bp fragment of the mitochondrial control region were carried out using primers LCM15382 and H950g [29]. PCR reactions were conducted in 20 µl volumes containing 10mM Tris, pH 8.4; 50 mM KCl, 0.5 µM of each primer, 1.5 mM MgCl_2_, 0.2 mM dNTPs, 0.5 unit of *Taq* DNA Polymerase, and approximately 10 to 30 ng of genomic DNA. PCR cycling parameters were as follows: 95°C for 3 min; 35 cycles of 95°C for 30 s, 55°C for 60 s, 72°C for 30 seconds; and a final extension of 72°C for 10 min. PCR products were purified by adding 2 µl of ExoSAP-IT® (USB Corporation) to 7 µl of the PCR reaction and incubated according to manufacturer’s instructions. The mtDNA amplicons were sequenced using ABI BigDye v3.1 (PE Applied Biosystems) and an ABI 3130*xl* or 3730*xl* DNA Analyzer with LCM15382 and H950g. Samples processed at the University of Georgia (Cape Verde) were sequenced using LCM15382 and an internal sequencing primer CC443, (TGATCTATTCTGGCCTCTG). Negative controls were included in each batch of PCR amplifications and sequencing reactions to detect contamination.

### Data Analysis

Sequences were aligned, edited, and compared to previously described haplotypes using the program Sequencher 4.2 (Gene Codes Corporation). Sequences were assigned haplotype designations after nomenclature published on the Archie Carr Center for Sea Turtle Research (ACCSTR) website (http://accstr.ufl.edu/accstr-resources/cclongmtdna.pdf). Original, short haplotypes received consecutive number designations based on the 380 bp sequence. Haplotypes based on the ∼800 bp fragment retain their original 380 bp designations and receive additional numeral suffixes to reflect any novel polymorphisms detected within the expanded sequences. Samples producing novel or ambiguous sequences were subjected to a second round of DNA extraction, PCR amplification, and sequencing for verification. Novel haplotypes were deposited with GenBank and ACCSTR. An unrooted parsimony network was created using the program TCS [30]. Haplotype distribution maps were generated using the Maptool function at www.seaturtle.org.

Population structuring at the study-wide scale was initially tested by considering the relative magnitude of barriers to gene flow as implemented in BARRIER based on frequency-based F_ST_ [31]. Haplotype diversity (*h*), pairwise F_ST_ comparisons, pairwise exact tests of population differentiation, and genetic distance-based analysis of molecular variance (AMOVA) were conducted using the software Arlequin version 3.5 [32]. Significance values for AMOVA were obtained from 10,000 permutations. Structure was examined using frequency-based AMOVA, frequency-based pairwise F_ST_ comparisons, and exact tests of population differentiation with p-values less than 0.05 considered significant. Exact tests of population differentiation were conducted with 100,000 permutations and 10,000 dememorization steps [33]. Correlation between genetic and geographical distances was determined using a Mantel test as implemented in Arlequin 3.5 [34]. Genetic distance was represented by F_ST_/(1-F_ST_), and geographical distances were untransformed shortest sea distances between rookeries that accounted for major continental coastlines [35].

Because only short sequences were available from Cuba and Tunisia, the reported F_ST_ values from comparisons involving Cuban and Tunisian haplotype profiles were generated from a separate analysis so that the differences between values generated from 380 and ∼800 bp haplotype data for remaining rookeries could be attributed solely to differences in haplotype resolution. Following pairwise F_ST_ comparisons and exact tests of population differentiation, proximal sample sites that were not significantly different were pooled for further analyses. Rookery clustering was also validated in an AMOVA framework by testing alternative management grouping scenarios in order to maximize F_CT_ and minimize F_SC_. To minimize bias in the case of incorrect pooling decisions, haplotype frequencies were weighted for each proposed management unit based on the relative size of individual rookeries comprising them (based on average nest counts or nest count ranges). Significance of the final round of pairwise F_ST_ comparisons and exact tests of population differentiation were adjusted using a false discovery rate correction with a table-wide α of 0.05 [36], [37].

Sequences were truncated to an 822 bp alignment for phylogenetic analyses including Pacific loggerhead turtle haplotypes and *Lepidochelys* as outgroups. Indels were coded as binary and included in analyses as a separate partition [38]. Two Pacific loggerhead turtle haplotypes for which comparable sequences were available from GenBank were included to provide phylogeographic context for Atlantic lineages: CCP1, the most common haplotype from eastern Australian rookeries [5], and CCP2, which is analogous to Japanese haplotype B and the most common haplotype among Japanese rookeries [39] (see Table S2 for GenBank accession numbers).

Divergence times were explored using Bayesian molecular clock frameworks implemented in BEAST v1.7.4 [40]. In order to calibrate nodes, control region sequences from Kemp’s ridley (*Lepidochelys kempii,* GenBank JX454981) and two olive ridleys (*Lepidochelys olivacea*) were included as outgroup taxa (GenBank AM258984 and JX454991). Fossil-derived divergence time spans at two nodes were used for calibration: the *Caretta-Lepidochelys* split at 12–20 million years before present (MYBP) [41], [42], and the *Lepidochelys olivacea-Lepidochelys kempii* split at 4.5 to 5 MYBP [43], [44]. Substitution model selection was conducted using MEGA5 [45]. A relaxed log-normal molecular clock was employed for the nucleotide partition, and the stochastic Dollo model was used for a partition consisting of binary indel data [46]. A chain length of 50,000,000 was used to ensure convergence and ESS values of at least 300 for all parameters.

## Results

### Haplotypes and Phylogeography

**Figure 2:**
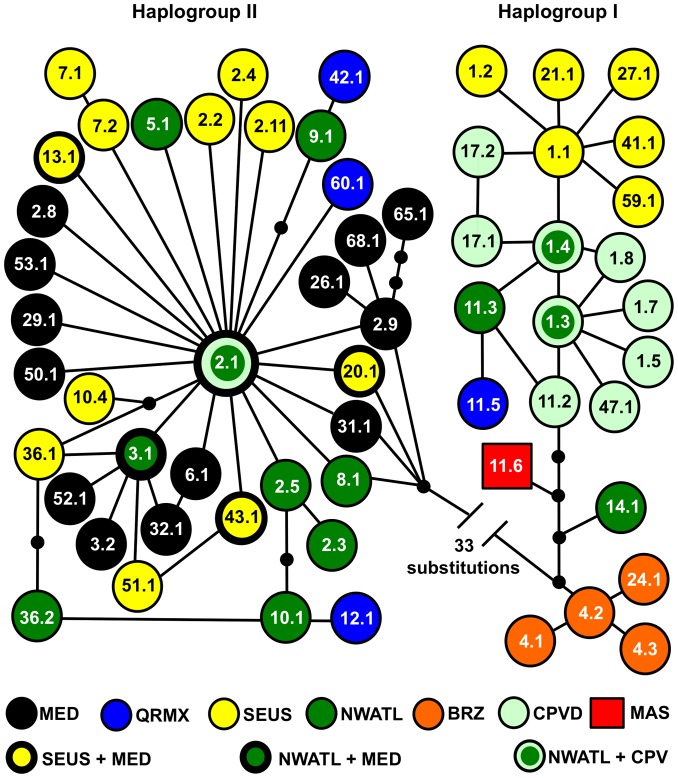
Unrooted parsimony network for ∼800 base pair Atlantic, Mediterranean, and western Indian Ocean loggerhead haplotypes. SEUS are southeastern United States rookeries. Small filled circles indicate inferred haplotypes that were not detected.

Excluding the Pacific haplotypes, 70 variable positions resolved 59 haplotypes for the expanded control region sequences (Table S2, Figure 2), which included 56 transitions, 11 indels, and 4 transversions. Position 530 contained both an indel and a transition. Short fragment haplotype CC-A4 from Brazil was subdivided into three variants: CC-A4.1, CC-A4.2, and CC-A4.3 (GenBank nos. EU179457, KF840724, and KF840725, respectively). The new samples from Boa Vista, Cape Verde yielded two new CC-A1 variants: CC-A1.7 and CC-A1.8 (GenBank KC310493 and KC310494), bringing the total to five CC-A1 variants recorded for Cape Verde and seven among North Atlantic rookeries. All individuals from South Africa carried the ubiquitous haplotype CC-A2.1, which has been previously documented from all Mediterranean rookeries and all Northwest Atlantic rookeries except for the northern MU [24]–[26], [28], [47]. All individuals from Oman were fixed for a novel variant of CC-A11 not previously detected among Atlantic rookeries (CC-A11.6, GenBank KF770994). Haplotype diversity varied from zero to 0.819 (Table S3). The highest haplotype diversity was recorded from mainland Quintana Roo (Mexico) rookeries, whereas the Southwest Indian Ocean RMU, Northwest Indian Ocean RMU, CAP and OSS rookeries in the Northwest Atlantic, and ALA in the Mediterranean were fixed for a single haplotype.

**Figure 3:**
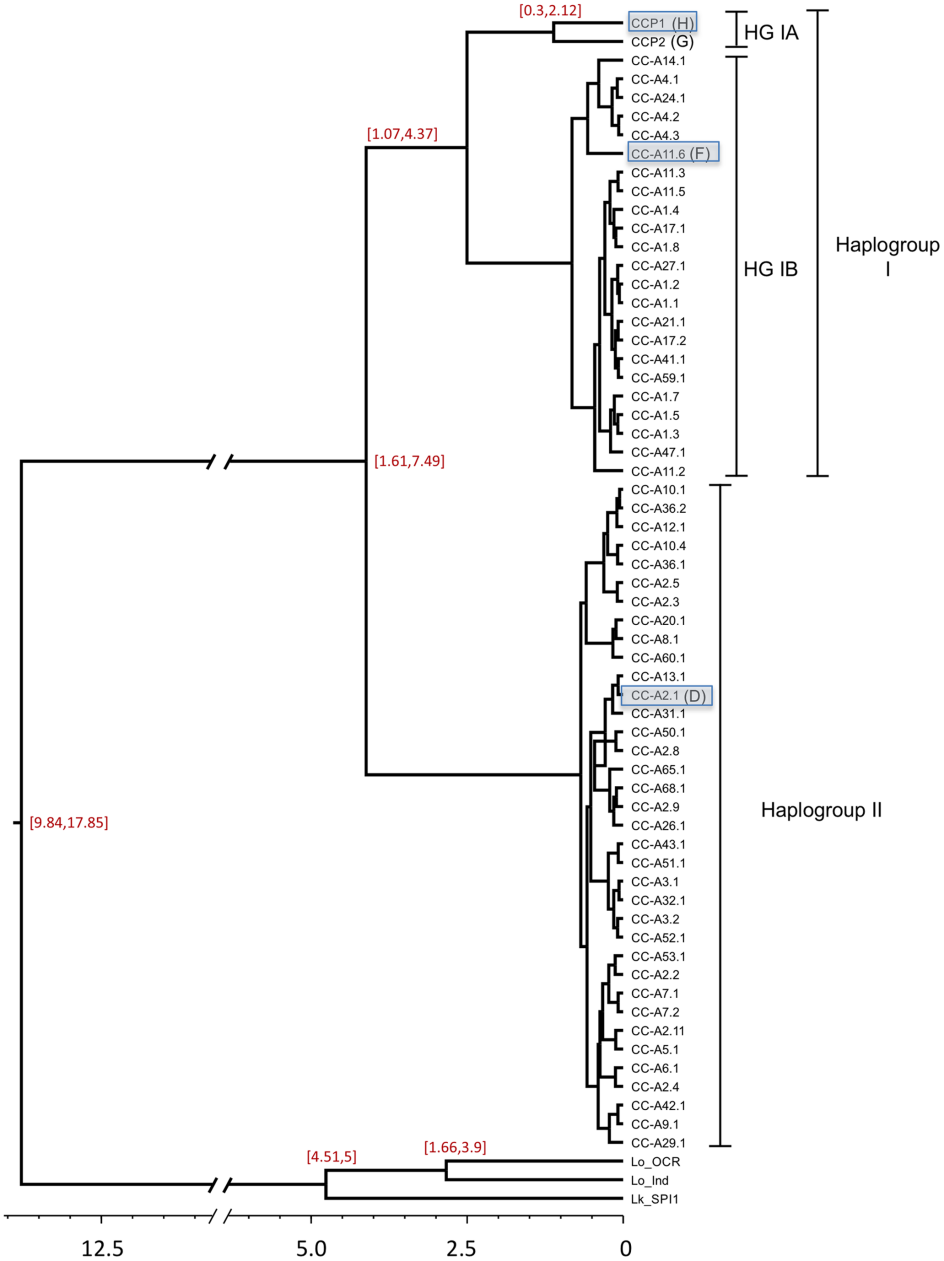
Chronogram for loggerhead turtle 800 base pair control region haplotypes based on a Bayesian relaxed-clock model as implemented in BEAST. The x-axis indicates divergence times in million years before present. 95% highest posterior density (HPD) intervals are indicated for major tree nodes. RFLP haplotype names [13] are included in parentheses beside their sequence-based haplotype designations. Haplotypes present in Indian Ocean rookeries are shaded in blue.

Bayesian phylogenetic analysis indicated the presence of two major loggerhead lineages globally (Figure 3). The deepest bifurcation among loggerhead lineages was estimated at 4.3 MYBP (95% highest posterior density (HPD): 1.6 to 7.5). Haplogroup II (containing CC-A2 haplotypes) was characterized by shallow structure relative to haplogroup I (containing CC-A1 haplotypes) (Figure 2). The deepest divergence among clade I lineages occurred between western Pacific/southeastern Indian Ocean haplotypes (haplogroup IA) and the remaining Atlantic and Indian Ocean haplotypes (haplogroup IB). This coalescent was dated at 2.7 MYBP (95% HPD: 1.1 to 4.4). A clade containing Brazilian haplotypes, Caribbean CC-A14, and CC-A11.6 from Oman diverged from the remaining haplogroup IB lineages approximately 1.0 MYBP (95% HPD: 0.3 to 1.3).

### Population Structure

Of 91 initial pairwise tests among Northwest Atlantic rookeries analyzed for longer sequences, 83 pairwise F_ST_ comparisons and 78 exact tests of population differentiation were significant (Table S4). Nearly all non-significant comparisons involved proximal sample sites within regions. These were pooled as follows for the second round of comparisons: CAP and OSS (NUSA); CAN and MEL (CEFL); JUN and FTL (SEFL); DRT and CSL (DRSL); ICZ and QRM (QRMX); and SGI and CSB (NWFL). The resulting six pooled MUs as well as KEY and CSK (Figure 1A) were all significantly different following FDR correction of the second round of comparisons (Table S5). Haplotype frequencies for SWC were significantly different from the remaining eight MUs based on 380 bp haplotypes for at least one of the F_ST_ comparisons or exact tests in the intermediate round of comparisons (Table S5) but not differentiated from QRMX following FDR correction of the final round of tests (Table S6).

In the initial pairwise tests comparing Brazilian rookeries, there was indication of structure between RIO and the rookeries to the north but not between the two central Brazilian rookeries (Table S4, Figure 1). Haplotype frequencies from the three northernmost Brazilian states were pooled for the final round of comparisons: NBRZ- SER/BAH/ESP. Following FDR correction, NBRZ vs. RIO comparisons were significantly different (Table S6). Despite lack of differentiation between central Brazilian rookeries based on ∼800 bp haplotype frequencies, we tested for a genetic break there based on the previous recommendation of recognition of northern and southern MUs [21]. Combined 380 bp haplotype counts from [21] and the present study supported a break between BAH and ESP (Table S7) with pooled northern (BRZN: SER and BAH) and southern (BRZS: ESP and RIO) rookeries well differentiated (F_ST_ = 0.101, p<0.00001; exact test p = 0.00002). Haplotype frequencies under a BRZN and BRZS pooling scenario (with a break between BAH and ESP) were also significantly different with 800 bp haplotypes (F_ST_ = 0.051, p = 0.027; exact test p = 0.00827).

In the initial round of comparisons for Cape Verde rookeries, there was evidence of structure between SAL and BOA as well as SAL and SLZ, but not among any of the remaining rookeries. However pooled haplotype frequencies under the clustering scenario of BOA/SLZ/MAO vs. SAL were not significantly different following FDR correction (F_ST_ = 0.027, p = 0.009; exact test p = 0.012), indicating a lack of strong structure among island rookeries comprising the Northeast Atlantic RMU (Figure 1C).

Among Mediterranean rookeries, the initial round of pairwise tests using long sequences indicated no differentiation among several proximal sample sets that were pooled for the second round of tests (Table S4, Figure 1B): ZAK, KYP, LAK (WGRC), AKA and ALA (CYPR), TKM and TKE (TKME); LEB and ISR (LBIS). Of 55 Mediterranean pairwise comparisons in the second round of tests, 24 pairwise F_ST_ comparisons and 15 exact tests of population differentiation were not significant following FDR correction (Table S5). Further clustering was warranted for DLY and DAL (DYDL), and MIS and SIR (LIBY). The final round of pairwise comparisons suggested seven distinct clusters: CAL, WGRC, CRT, DYDL, TKW, LIBY, aside from the remaining eastern basin rookeries (EMED): TKME/LBIS/CYPR. TUN was not significantly different from the proximal Libyan rookeries based on short haplotype sequences (Table S6).

Application of up to ten barriers in the BARRIER analysis differentiated among the six RMUs as well as indicating structure within the Northwest Atlantic and Mediterranean RMUs. The Mantel test detected the presence of very weak but significant isolation by distance (correlation coefficient = 0.105, p = 0.002). There was significant differentiation among discrete rookeries based on expanded haplotype frequencies (F_ST_ = 0.349, p<0.0001). The geographic distribution of haplotype frequencies among RMUs accounted for the majority of genetic partitioning, although additional structuring among rookeries within some of the RMUs was evident, particularly within the Northwest Atlantic RMU (Table S8). Grouping of Mediterranean rookeries was tested in an AMOVA framework by alternative rookery clustering scenarios while holding 14 discrete and strongly supported RMUs and MUs constant (Table S8). Thus, the overall pairwise and AMOVA analyses supported the genetic distinction of the Northeast Atlantic, Southwest Indian, and Northwest Indian Ocean RMUs as well recognition of 18 additional MUs within the remaining RMUs considered in the analysis: **Northwest Atlantic RMU**- 1) NUSA, 2) CEFL, 3) SEFL, 4) DRSL, 5) QRMX/SWC, 6) KEY, 7) CSK, 8) NWFL; **Southwest Atlantic RMU**- 8) BRZN, 9) ESP, 10) RIO; **Mediterranean Sea RMU**- 11) CAL, 12) WGRC, 13) CRT, 14) DYDL, 15) TKW, 16) EMED, and 17) LIBY/TUN.

## Discussion

### Phylogeography

Previous restriction fragment length polymorphism (RFLP) analysis of the mitochondrial genome indicated two deeply divergent global lineages among loggerhead turtles. One major haplogroup was represented by RFLP haplotype B (now sequence CC-A1) from the southeastern USA, RFLP C (sequence CC-A4) from Brazil, and RFLP F (sequence CC-A11) from Oman. The second major haplogroup was represented by RFLP haplotype D (sequence CC-A2) from the southeastern USA, South Africa, and Greece; RFLP G from Japan (sequence CCP2 and CCP3), and RFLP H (sequence CCP1 and CCP5) from Australia [13]. In a more recent analysis based on 380 bp control region sequences, the western Pacific lineages (CCP sequences) clustered more closely with the clade containing CC-A1 rather than CC-A2 [48], consistent with the results of the present study. A major divergence between haplogroups I and II followed by a more recent split of haplogroup I lineages (Figure 3) is also congruent with a mitogenomic phylogeny that included three global haplotypes (CC-A1, CC-A2, CCP1) [49]. The point estimate of a primary bifurcation approximately 4.1 MYBP generated from the present study is congruent with the coalescence time for loggerhead lineages generated from mitogenomic phylogenetic analysis [49]. Point estimates in both studies fall at the upper limit of the divergence time range inferred from RFLP analysis (2 to 4 MYBP), but these estimates are not inconsistent with the previous hypothesis that the rise of the Isthmus of Panama split the ancestral lineage [13], particularly given the broad HPD ranges of the latter studies.

The CCP haplotypes (haplogroup IA) currently found in eastern Indian and western Pacific Ocean rookeries appear to be the oldest extant lineages in the species having diverged from the remaining clade I lineages approximately 2.7 MYBP. An Indo-Pacific origin for at least one of the major loggerhead turtle clades would be consistent with phylogeographic scenarios proposed for other marine turtle species. Analysis of global ridley turtle (*Lepidochelys*) haplotypes suggested that the northern Indian Ocean represented the ancestral refugium from which all extant lineages radiated [50], [51]. Similarly, global analysis of control region sequences indicated the Indo-Pacific as the likely source of the most recent radiation of leatherback turtles (*Dermochelys coriacea*) that currently nest circumglobally [52].

The presence of CC-A2.1 at high frequencies among three RMUs spanning the western Atlantic Ocean, Mediterranean Sea, and Southwest Indian Ocean basins suggests a relatively rapid colonization sequence or rapid demographic expansion of newly founded rookeries in these regions. By contrast, structuring of haplogroup IB lineages could be defined by radiations from a small number of basal haplotypes (Figure 2), implying *in situ* diversification and stepping stone colonization at each of the three Atlantic RMUs containing haplotypes in this clade. Based on analyses of 380 bp haplotypes, Reis et al. hypothesized that individuals from the large southeastern United States aggregation could have founded the Brazilian nesting aggregation given the relationship of CC-A1 and CC-A4 [21]. However, with expanded sequences it is clear that the Brazilian haplotypes appear basal among haplogroup IB lineages, thus the Brazilian rookery harbors lineages older than those nesting in the southeastern United States. CC-A14 likely represents a relict Caribbean lineage given that it is most common in the Cuban rookeries [53] and occurs at low frequency elsewhere in the Northwest Atlantic RMU [28]. CC-A1.3, the dominant haplotype at Cape Verde, forms the center of a star-like radiation of haplotypes and appears more derived than the CC-A4 lineages (Figure 2). This suggests that Cape Verde may host the second oldest extant loggerhead turtle rookery in the Atlantic. The most widespread haplogroup I haplotype in the United States rookeries is CC-A1.1. Its derived position relative to CC-A1.3 and CC-A1.4 implies that the United States hosts the youngest haplogroup I lineages in the Atlantic despite the fact that this nesting aggregation is the largest in the Atlantic basin [28]. CC-A11 variants were quite rare among North Atlantic rookeries (∼0.28%) and were well differentiated from the Indian Ocean variant.

Colonization of novel nesting beaches often occurs “down current” of established rookery sites and in the vicinity of developmental foraging areas, highlighting the potential importance of dispersal during the oceanic stage for facilitation of rare long-range colonization events [54]. Proposed invasions of the Mediterranean by lineage(s) of Atlantic origin [24], [55] are consistent with this scenario given that oceanic juvenile turtles of western Atlantic origin are known to enter the Mediterranean [56]. Bowen et al. proposed a similar scenario for colonization of the Atlantic by the RFLP haplotype D lineage (now confirmed as CC-A2.1) given the “leakage” of oceanic juveniles of South African origin into the South Atlantic [13]. Recoveries of notched oceanic juveniles have indicated that most hatchlings emerging from beaches in South Africa enter the Agulhas Current with a portion of these being swept into the South Atlantic [57], thus confirming that the warm water eddies of the Agulhas Current serve as a transport mechanism for turtles from the Indian Ocean into the Atlantic.

**Figure 4:**
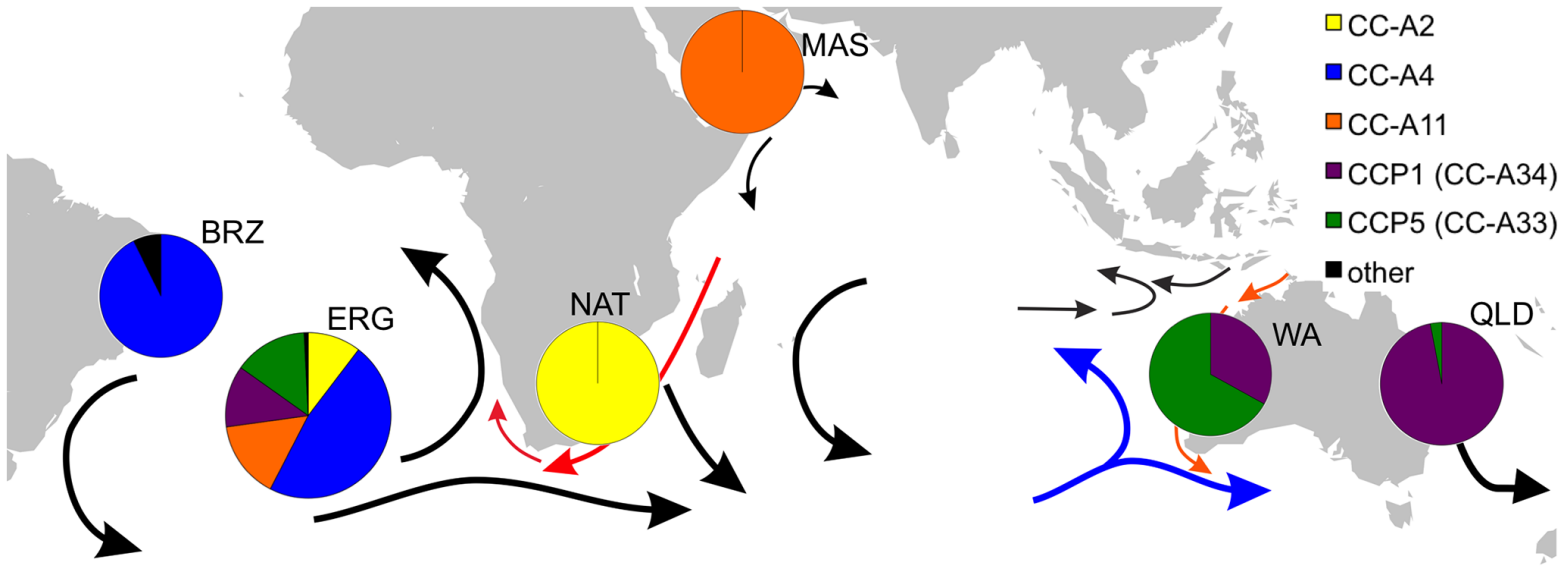
Loggerhead turtle haplotype distribution for an oceanic foraging aggregation and major Indian Ocean rookeries. Control region haplotype (380 base pair) frequencies for the oceanic juvenile foraging aggregation from the Elevação do Rio Grande seamount (ERG) and adjacent ridge and slope of the continental shelf in the South Atlantic Ocean [21] and the RMUs in the South Atlantic, Indian, and South Pacific Ocean basins. BRZ is combined Brazilian rookeries [21]; NAT is Natal, South Africa (present study); MAS is Masirah Island, Oman (present study); WA is Western Australia [58], QLD is Queensland, Australia rookeries [5]. The arrows indicate directionality of major surface currents. Orange represents the Leeuwin Current. Blue indicates the Western Australia Current. The Agulhas current is highlighted in red.

An additional inference of possible connectivity between the Indian and South Atlantic basins comes from MSA of oceanic juvenile loggerhead turtles incidentally captured by the longline fisheries operating in the South Atlantic off the coast of Brazil. Aside from CC-A4 juveniles that could be assigned confidently to the Brazil rookeries, four other haplotypes dominated the foraging aggregation (CC-A2, CC-A11, CC-A33, CC-A34; [21]) (Figure 4). CC-A33 is identical to CCP5 (ACCSTR reference sequence and GenBank EF033112, respectively), and CC-A34 is identical to CCP1 (ACCSTR reference sequence and GenBank EF033112, respectively). These haplotypes are only known to co-occur in the rookeries of Western Australia, Queensland, and New Caledonia [5], [58], and the relative frequencies of the two haplotypes in the foraging aggregation are roughly concordant with their frequencies in the Western Australian rookery. However, investigators cautioned that haplotypes of apparent Australian origin may represent unsampled rookeries [21]. Dispersal patterns for oceanic juveniles modeled under the assumption of passive drift supported the likelihood of dispersal of South African juveniles into the South Atlantic but did not indicate connectivity between the South Atlantic and more distant rookeries in the Indian Ocean basin [59]. Nonetheless, confirmation of CC-A2.1 from South Africa and CC-A11.6 from Oman in the present study warrants reconsideration of the possibility of long distance migratory connectivity between the South Atlantic and Indian Ocean basins. The four haplotypes present in the three RMUs spanning the Indian Ocean comprised nearly half of the South Atlantic foraging aggregation (Figure 4), and all known significant loggerhead turtle rookeries in the Indian and Atlantic Oceans have been genetically characterized.

Migratory connectivity on this scale is consistent with oceanic juvenile dispersal linking Japanese rookeries with foraging sites in Pacific Mexico [6] and eastern Australian rookeries with foraging sites off the coast of Peru [5]. However, dynamics of the Indian Ocean subtropical gyre and specifically the eastern boundary current in the southeastern Indian Ocean are more complex than those of the major Pacific basin gyres. Observations of post hatchling loggerhead turtles washed ashore along the southwestern and southern coasts of Western Australia following storms suggest the Leeuwin Current transports at least some turtles southward from their natal rookeries [60]. However, genetic analysis of oceanic juveniles stranded along the southeastern Australian coast and in New Zealand indicate an eastern Australian origin [5], suggesting that juveniles of Western Australian origin do not reach the eastern coast. The strength of the Leeuwin Current varies interannually with the El Niño-Southern Oscillation as well as seasonally, with weaker flows in the summer months [61]. The Leeuwin Current frequently sheds warm core eddies, several of which are sufficiently long-lived to reach the West Australian current that forms the eastern arm of the Indian Ocean subtropical gyre [62], invoking the possibility of broad scale Indian Ocean connectivity that warrants further investigation. Although we echo the caveat of previous investigators that haplotypes CC-A33 and CC-A34 could be originating from genetically uncharacterized rookeries (possibly in western Africa or elsewhere in the Indian Ocean), this scenario would still suggest broad scale connectivity between the ocean basins in an evolutionary context. Further, if South Atlantic oceanic juveniles with haplotypes CC-A33 and CC-A34 do not originate from Western Australia, their relatively large proportions in the aggregation (∼26%) [21] would indicate the existence of an as yet undescribed globally significant loggerhead turtle rookery or rookeries.

Invasion of the Atlantic by Indo-Pacific lineages of marine organisms via southern Africa is well established [63], and is supported in this study in the case of the haplogroup I loggerhead lineage. However, patterns of haplotype diversity suggest that recent colonization of South Africa by an Atlantic CC-A2 lineage is more likely than an east to west colonization, despite the presence of South African oceanic juveniles in the South Atlantic. The South African rookery only contains CC-A2.1 whereas at least 30 haplotypes derived from it have been recorded from western Atlantic and Mediterranean rookeries. A similar phylogeographic pattern occurs in green turtles (*Chelonia mydas*) where haplotype CM-A8 is the sole representative of its clade in South Mozambique Channel rookeries in the Southwest Indian Ocean [64], whereas a large number of haplotypes apparently derived from CM-A8 occur along with it in South Atlantic rookeries [65], [66]. Recent colonization of Europa and Juan de Nova by a lineage of South Atlantic origin was implicated to explain the observed distribution of haplotypes and the lack of unique haplotypes descended from CM-A8 in the Indian Ocean [64]. An alternative hypothesis is that the CC-A2.1 lineage that gave rise to the loggerhead Atlantic and Mediterranean haplogroup II lineages was retained in the Indian Ocean. Such a scenario would suggest recent demographic expansion following a severe bottleneck to explain the lack of haplotype diversity in the South African rookery. Mitogenomic sequences and nuclear markers may facilitate testing of the alternative east to west and west to east hypotheses.

Inter-oceanic exchange of loggerhead turtle lineages likely occurred multiple times [13], [48]. Colonization in both directions likely occurred in haplogroup I lineages whereby the ancestor of present-day Atlantic lineages invaded from the Indo-Pacific followed by much more recent colonization of the Indian Ocean from the Atlantic by the precursor of CC-A11.6, given its nested position among Atlantic haplotypes. Colonization from the Atlantic into the Indian Ocean via the Cape of Agulhas appears quite rare relative to the opposite pattern. In addition to green turtles [64], gene flow from the Atlantic into the Indian Ocean has been proposed for scalloped hammerhead sharks (*Sphyrna lewini*) [3]. The possible recent invasion of the Indian Ocean from the Atlantic by at least three different marine turtle lineages suggests that colonization of novel nesting sites distant from natal rookeries is much more complex than a breakdown of natal homing mechanisms in the presence of oceanic dispersal of small juveniles.

### Population Structure

Haplotype compositions for marine turtle rookeries are widely used for two primary purposes: 1) to inform recognition of demographically partitioned rookery MUs for the purposes of conservation and management, and 2) to provide baseline frequencies for MSA of foraging aggregations. Ideally, these would be accomplished via a singular dataset, but even with the current expanded sequences, marker resolution remains a significant impediment to fully realizing this goal. Furthermore, investigators may be conducting MSA at varying spatial scales and in different regions, which may shift priorities for rookery splitting and lumping. For example, analysis of juvenile loggerheads in the western Mediterranean may be conducted hierarchically, first assigning Atlantic versus Mediterranean origins for all individuals where possible (see [71]) followed by discrete analysis of specific Mediterranean MU contributions to that portion of the dataset. Similarly, an analysis of oceanic juveniles in the South Atlantic would likely target specific Brazilian MU contributions, whereas all Brazilian MUs might be clustered as a single unit for the purposes of assigning stocks for juveniles from the North Atlantic where Brazilian turtles are quite rare (eg. [20]).

Previous analysis of 380 bp haplotypes indicated that the Brazilian nesting aggregation could be divided into northern (Sergipe and Bahia) and southern (Espírito Santo and Rio de Janeiro) MUs based on the presence/absence of haplotype CC-A24 [21]. However, subdivision of CC-A4 in the present study suggests that the strongest demographic partitioning within the Brazilian nesting aggregation may occur between the rookeries of Rio de Janeiro state relative to all others in the nation. Analyses based on the 380 bp haplotypes and using the combined sample sets from [21] and the present study support recognition of northern and southern Brazil MUs with a break between BAH and ESP. Given the combination of 380 bp and ∼800 bp results with different sample sets, we tentatively propose the recognition of three MUs within the Southwest Atlantic RMU: northern coast (Sergipe and Bahia), Espírito Santo, and Rio de Janeiro. In light of the subdivision of CC-A4 obtained with the expanded control region fragments, analysis with larger sample sizes from the Brazilian rookeries is warranted to better resolve the number of MUs and their boundaries.

Observed genetic differentiation supported the genetic distinctiveness of the Northeast Atlantic, Southwest Indian, and Northwest Indian Ocean RMUs and the presence of at least 18 MUs across the remaining RMUs considered in this study, defining 21 population units for loggerhead turtles globally exclusive of eastern Indian Ocean and western Pacific Ocean rookeries. Within the Northwest Atlantic RMU, eight rookery clusters were evident: 1) northern MU (NUSA), 2) central eastern Florida (CEFL), 3) southeastern Florida (SEFL), 4) Cay Sal, Bahamas and the Dry Tortugas, Florida (DRSL), 5) Quintana Roo, Mexico (QRMX) and SWC, 6) southwestern Florida (KEY), 7) central western Florida (CSK), and 8) northwestern Florida (NWFL). Within the Mediterranean RMU, there was support for at least seven rookery clusters: 1) Calabria, Italy (CAL), 2) Libya (LIBY), 3) western Greece (WGRC), 4) Crete (CRT), 5) Dalyan and Dalaman, Turkey (DLY/DAL), 6) western Turkey (TKW), 7) and the remaining eastern basin rookeries (TKM/TKE/ALA/AKA/LEB/ISR). No structure was detected among islands comprising the Northeast Atlantic RMU, and no genetic variation was detected among individuals within the Southwest Indian Ocean and Northwest Indian Ocean RMUs. Sampling effort should be expanded for Oman to further explore haplotype diversity given the small sample size and the vast numbers of females that nest there [57].

Recognition of additional MUs for the purposes of rookery management may be necessary. This is particularly true in the Mediterranean RMU, where CC-A2.1 dominated the haplotype profiles for all rookeries except DAL. Given the scale of genetic structure evident among the westernmost rookeries in the Mediterranean basin, middle and eastern Turkey, Cyprus, and the coast of Lebanon/Israel likely represent demographically independent rookeries relative to one another and should be managed accordingly despite the lack of observable genetic structuring among them. Frequencies of 380 bp haplotypes for the Tunisian and Libyan rookeries were not significantly different, but several hundred kilometers of separation would suggest that these nesting populations are demographically isolated. Given the presence of CC-A2.9 in the Libyan rookeries, reanalysis of Tunisian samples using the longer sequences should be a high priority. At finer spatial scales, the two Libyan rookeries and DLY and DAL in Turkey were not significantly different following FDR correction, but this apparent lack of strong differentiation should be revisited with larger sample sizes and additional markers in future studies given that p-values approach significance. Recognizing the distinction of these four rookeries pushed the F_SC_ value from significant to insignificant in the AMOVA (Table S8). In the Northwest Atlantic RMU, SWC was not significantly different from QRMX in the present analysis following FDR correction. However, this comparison was significantly different when analyzed at the RMU level only [28], and we continue to advocate for separate management of these rookeries pending additional analyses of the Cuban dataset. Similarly, CSL and DRT were not significantly different based on haplotype frequencies, but Shamblin et al. [28] suggested that they be recognized as demographically distinct MUs based on their discreteness and isolation given the overall pattern and scale of structure inferred among rookeries elsewhere in the nesting aggregation.

### Collaborative Model for Future Research

The 800 bp haplotype frequency data contributed by this study provide a baseline for improved resolution in future MSAs. The indication of apparently diagnostic differentiation between Indian and Atlantic Ocean CC-A11 using the expanded control region sequences has important implications for determining the rookery origins of South Atlantic oceanic juveniles, and reanalyzing Brazilian oceanic juvenile samples [21] for the longer control region fragment should be a high priority. Similarly, the variation uncovered in CC-A4 from Brazil should be further explored and permits the possibility of testing for juvenile natal homing along the Brazilian coast as has been demonstrated in the southeastern United States [8]. Despite the gains in stock resolution realized with the expanded control region fragment, most CC-A2 individuals nesting in the Northwest Atlantic and Mediterranean RMUs and all individuals screened from the Southwest Indian Ocean RMU remain confounded as CC-A2.1. This widespread haplotype sharing will undoubtedly weaken inferences from MSA, and exploration of additional genetic markers to tease out informative variation is needed.

Expanded effort with nuclear markers has demonstrated that the paradigm of weaker structure inferred from nuclear versus mtDNA in marine turtles, attributed at least in part to male-mediated gene flow [4], is not universal [67], [68]. Microsatellite analyses detected demographic isolation of rookeries that were undifferentiated with respect to mtDNA haplotype frequencies in Mediterranean green turtles [69] as well as Atlantic leatherbacks [70]. Although southeastern United States loggerhead turtle rookeries exhibit essentially no structure at microsatellite loci [15], microsatellites have identified demographic partitioning among Mediterranean rookeries, some of which were undifferentiated with respect to mtDNA [22]. Further, most turtles carrying haplotype CC-A2.1 and foraging in the Mediterranean could be confidently assigned to Atlantic or Mediterranean rookeries via assignment tests based on microsatellite allele frequency differences [71]. This study also predicted that CC-A21.1 and CC-A27.1 would be of Atlantic origin through use of Atlantic versus Mediterranean microsatellite baseline frequencies, a conclusion that Shamblin et al. confirmed [28]. Microsatellite allele frequency partitioning was also previously demonstrated between Northwest Atlantic and Southwest Atlantic loggerhead rookeries [15]. It is therefore likely that the six RMUs included in this analysis are isolated with respect to nuclear gene flow, but this remains to be tested through more global application of microsatellites and/or single nucleotide polymorphisms. Nuclear markers may be particularly helpful in confidently assigning rookery origins for South Atlantic oceanic juveniles, as has been demonstrated for Atlantic leatherback turtles where MSA based solely on mtDNA haplotypes could not fully resolve source rookery assignments [72].

Mitogenomic sequencing may resolve some cases of haplotype sharing across rookeries. The mitogenomes of 20 green turtles that shared common 490 bp control region CM-A5 revealed five variable positions resolving four haplotypes that were regionally partitioned among Greater Caribbean rookeries and provided the first genetic evidence of differentiation among eastern Caribbean rookeries [73]. Mitogenomic sequencing of CC-A2.1 loggerhead turtles from the Northwest Atlantic, Mediterranean, and South African rookeries may yield MU and RMU informative variable positions, which would potentially improve assessments of stock structure and phylogeography as well as simplify MSAs with respect to the current requirement of data from multiple nuclear markers. Indeed, four variable positions were evident in alignments of the two CC-A2.1 mitogenomes in GenBank representing Northwest Atlantic and Greek rookeries (JX454983 [49] and FR694649 [74], respectively), and these positions should be screened for potential utility as markers in MSAs. Beyond variable positions, a repetitive element in the control region has been identified as potentially informative for fine scale analyses of structure in loggerhead and green turtles [56], [74], [75], although homoplasy may render the marker less effective across large scale (among RMU-level) comparisons for loggerheads [56].

The working group has provided a framework for cooperation and coordination among research groups, sharing data prior to publication, and highlighting key areas for future research. Although the database we present here is the most comprehensive to date, it will require continual updating as additional rookery data become available to maintain relevance for MSA in the future. We have established a website to provide “live” web-based forum for obtaining an updated version the haplotype frequencies (Table S3). Further refinements of stock structure through exploration of novel genetic markers and fully realizing the benefits of increased resolution in MSA for this highly migratory species will require continued collaboration across ocean basin and global scales. Genetic studies have informed several aspects of marine turtle life history, and within the working group framework, genetic tools are poised to continue to make significant contributions to the conservation of this species.

## Supporting Information

Table S1 **Metadata and sampling codes for Atlantic, Mediterranean, and Southwest Indian Ocean loggerhead turtle rookeries.**
(XLS)

Table S2 **Variable positions for expanded control region loggerhead turtle haplotypes recorded from global rookeries.**
(XLS)

Table S3 **Mitochondrial DNA control region haplotype counts based on 800 bp sequences and haplotype diversity for loggerhead turtle rookeries in the Mediterranean Sea, Atlantic, Southwest Indian Ocean, and Northwest Indian Ocean basins.**
(XLS)

Table S4 **Pairwise comparisons based on 800 base pair (bp) control region haplotype frequencies for loggerhead turtle rookeries in the Mediterranean Sea, Atlantic, Southwest Indian Ocean and Northwest Indian Ocean.**
(XLS)

Table S5 **Pairwise comparisons based on 800 base pair (bp) control region haplotype frequencies for pooled loggerhead turtle rookeries in the Mediterranean Sea, Atlantic, Southwest Indian Ocean and Northwest Indian Ocean (following an initial round of comparisons).**
(XLSX)

Table S6 **Pairwise comparisons of RMU and proposed MU haplotype frequency differentiation based on 800 bp haplotypes.**
(XLSX)

Table S7 **Pairwise comparisons of 380 bp haplotypes from Reis et al. 2010 and the present study for Brazilian loggerhead rookeries.**
(XLS)

Table S8 **AMOVA results for alternative management scenarios to test Mediterranean groupings based on 14 core RMUs and MUs that were held constant in their isolation: 1) NUSA, 2) CEFL, 3) SEFL, 4) DRSL, 5) QRMX, 6) KEY, 7) CSK, 8) NWFL, 9) BRZN, 10) RIO, 11) CPVD, 12) CAL, 13) NAT 14) MAS.**
(XLS)
